# Patient’s safety culture among Tunisian healthcare workers: results of a cross sectional study in university hospital

**DOI:** 10.11604/pamj.2016.24.299.8466

**Published:** 2016-08-03

**Authors:** Asma Ben Cheikh, Nabiha Bouafia, Mohamed Mahjoub, Olfa Ezzi, Amel Nouira, Mansour Njah

**Affiliations:** 1 Hospital Hygien Service, University Hospital Center Farhat Hached, Sousse, Tunisia

**Keywords:** Patient safety, perception, health professionals, Tunisia

## Abstract

**Introduction:**

Healthcare safety has become a public health priority in developed world. Development of safety culture care is fundamental pillar to any strategy for improving quality and safety care. The objective of this study is to measure level of patients’ safety culture among healthcare professionals at university hospital, center Farhat Hached Sousse (Tunisia).

**Methods:**

We conducted, in 2013, a descriptive study among all licensed physicians (n= 116) and a representative sample of paramedical staff (n= 203) exercising at university hospital center Farhat Hached Sousse (Tunisia). Measuring instrument used is a valid questionnaire containing ten safety care dimensions. Data were analyzed using SPSS version 19.

**Results:**

The response rates were 74.1% for physicians and 100% for paramedical staff. Overall score of different dimensions varies between 32.7% and 68.8%. Dimension having most developed score (68.8%) was perception of “Frequency and reporting adverse events”. Dimension with lowest score (32.7%) was “Management support for safety care”.

**Conclusion:**

Our study has allowed us to conclude that all dimensions of patients’ safety culture need to be improved among our establishment’s professionals. Therefore, more efforts are necessary in order to develop a security culture based on confidence, learning, communication and team work and rejecting sanction, blame, criminalization and punitive reporting.

## Introduction

Healthcare safety has become a public health priority in developed world [1]. In fact, developing safety culture (SC) could decrease the number of adverse events (AEs) in healthcare [1, 2]. Indeed, AEs still remain as a global challenge and no country has yet overcome all of its patient safety problems [3]. So, many studies have shown the severity of these accidents, both in terms of cost, frequency and serious consequences [1]. The overall incidence of AEs in various developed countries varies between 2.9% and 16.6% [2, 4]. Another report mentioned that 44,000 to 98,000 people die due to medical errors in the United States every year [1, 5]. The situation is more difficult and serious in developing countries with higher risk of patient harm due to the limitation of resources and lack of adequate infrastructures [3]. In fact, AEs are unacceptably among the five most common causes of preventable death and millions of patients are hurt each year due to unsafe care practices [3]. Therefore, many countries, particularly industrialized, have developed national strategies to reduce the incidence of these AEs [1, 6]. One of these strategies is the development of patient SC. In fact, the success of any intervention with the ultimate goal of securing care and reducing AEs must go through the development of a patient SC with healthcare workers [1, 6]. So, Nieva and Sorra defined patient SC as a product of individual and group values, attitudes, perceptions, competencies, and patterns of behavior that determine the commitment to and the style and proficiency of an organization´s health and safety management [4, 7]. In Tunisia, the incidence of serious AEs varied between 10 and 11.3% (95% CI {9.6 - 12.9}) [8]. Aware of the relatively high frequency and severity of this complication, we have established an action plan for the prevention in order to improve patient safety within the health care units of university hospital center (UHC) Farhat Hached, Sousse - Tunisia. The objective of this study, is to identify the sources and degree of information, to measure the level of patient SC among healthcare workers in UHC Farhat Hached in order to detect failures and to solve the problems in the hospital related to patient safety.

## Methods

### Study setting

Data was collected from 16 units of the Farhat Hached UHC (Sousse-Tunisia). This hospital is composed of 26 medical units, 4 surgical units, 9 laboratories and has a capacity of 698 beds in 2012. It includes 1354 health workers, 220 doctors, dentists and pharmacists and 1134 paraclinical personnel. Ethical approval for the study was obtained from all participants.

### Study design and sampling

A cross-sectional descriptive study was conducted among health professionals of the hospital during two months (June and July 2012). The population of the study consisted of all physicians (n=116) and a representative sample of paramedical staff (nurses and superior technicians) (n=203) working in the 16 units of University Hospital. Participation was anonymous, and participants were provided with an informational coversheet stating the aim and highlighting the voluntarily and confidential nature of the study results.

### Study survey

Data were collected using a survey inspired from Hospital Survey on Patient Safety Culture (HSOPS). The HSOPS was developed by the Agency for Healthcare Research and Quality (AHRQ) in 2004 and has been translated into around 20 different languages [3, 5]. It was translated into French and was validated by the Committee for Coordinating Clinical Evaluation and Quality in Aquitaine (CCECQA) [9, 10]. The French version of HSOPS was designed to measure 10 dimensions of patient SC (Overall perceptions of safety, frequency of event reporting, Supervisor/manager expectations and actions promoting safety, organizational learning and continuous improvement, teamwork within hospital units, communication openness and non-punitive response to error, staffing, hospital management support for patient safety, healthcare worker-patient relationship and patient SC, teamwork across hospital units). Each of these dimensions was composed of several items. Therefore, our questionnaire included 53 items related to patient safety, which uses the 4 point Likert response scale of agreement (“Strongly disagree” to “Strongly agree”): 48 items grouped into 10 defined patient culture security dimensions; 4 items related to sources of information on SC; one item concerned the perception of the results of patient safety level. For these items, were added 6 questions related to socio-demographic characteristics of participants.

### Statistical analysis

Data were analyzed using SPSS version 19. The percentage of positive responses for each item was calculated. For positively worded items, percent positive response is the percentage of respondents who answered “Strongly agree” or “Agree”. For negatively worded items, percent positive response is the percentage of respondents who answered “Strongly disagree” or “Disagree”. For each dimension, a score was calculated. It was the mean of the percentages of positive answers to the dimension’s respective items. A score equal or higher than 75 was considered to reflect a positive perception of the respondent towards the scored dimension. A score lower than 50 was considered to reflect a negative perception of the respondent towards the scored dimension.

## Results

In the following sections, we first show the demographic characteristics of respondents taking the survey. Next, we identified the sources and degree of information, and finally we measured the level of patient safety culture among healthcare workers. In total, of 319 healthcare personnel who participated in the study, 289 completed the questionnaire. The response rates were 74.1% (86/116) for doctors and 100% (203/203) for paramedical staff. The average age of the respondents was 38.3 ± 9.9 years old, and most of them were female (65.1%). 42.2 % of respondents had more ten years of experience at the hospital. The demographic characteristics of the final sample are described in Table 1. Forty-one point tow percent (41.2%) of respondents judged they were informed about the culture of patient safety. All of them claimed that the main source of information on the SC was firstly their experience (50.5%), followed by medical school (37.7%) and finally the general culture (25.8%) and media (22%). The perception of the doctors and paramedical staff on patient SC were summarized in Table 2. The global percentage of positive responses was highest for ‘Frequency of event reporting’ (68.8%), ‘Supervisor/manager expectations and actions promoting safety’ (68.1%) and lowest for ‘Hospital management support for patient safety’ (32.7%). The responses to the questions related to the difference of perception between doctors and paramedical staff on patient SC were summarized in Table 2. Concerning doctors, the dimensions that received lower positive response rate were ‘Hospital management support for patient safety’ (13.9 %) and ‘Teamwork within units’ (45.4 %), while those with highest positive response included “Supervisor expectations and actions promoting safety” (82.3%) and “Frequency of event reporting” (84%). For paramedical staff, the dimensions with the highest positive response were “organizational learning and continuous improvement” (67.8%), the lowest positive response was related to “Hospital management support for patient safety” (40.9%).

**Table 1:** Demographic characteristics of respondents, (N=289)

	n	%
**Staff position**		
Doctors	86	29.7
Paramedical staff	203	70.3
**Gender**		
Male	101	34.9
Female	188	65.1
**Working time in hospital**		
Less than ten years	122	42.2
More than ten years	167	57.8
**Work Unit/Department**		
Doctors	14	16.3
Surgery^a^	6	7
Obstetrics	57	66.3
Medicine^b^ Intensive care unit	9	10.4
Paramedical staff	54	26.6
Surgery^a^	46	22.6
Obstetrics	91	44.8
Medicine^b^ Intensive care unit	12	6

a Include units of abdominal surgery, ophtalmology and Oto-rhino laryngology

b Include units of internal medicine, pediatrics, carcinology, cardiology, dermatology, endocrinology, hematology, pneumology, rheumatology and psychiatry.

**Table 2:** Perception of the doctors and paramedical staff on patient safety culture

Dimensions	Score (%)		
	Global	Doctors	Paramedical staff
1- Overall perceptions of patient safety	61.1	63.8	60
2- Frequency of event reporting	68.8	84	62
3- Supervisor/manager expectations and actions promoting safety	68.1	82.3	62
4- Teamwork within hospital units	58.3	63.3	56
5- Teamwork across hospital units	48.6	45.4	50
6- Staffing	54.7	55.2	54.5
7- Communication openness and Non-punitive response to error	60.5	65.2	58.5
8- Hospital management support for patient safety	32.7	13.9	40.9
9- Healthcare worker – patient relationship and patient safety culture	58.4	69.7	53.4
10- Organizational learning and continuous improvement	67.9	68.2	67.8

## Discussion

This study describes the patient SC among healthcare workers in UHC Farhat Hached Sousse, Tunisia. The strength of the study was the higher rate of participation (90.5%). In fact, most of doctors (74.1%) and all of paramedical staff (100%) were surveyed and the literature suggests that response rates of more than 60% are adequate [11]. Therefore, this research, which reflect views of healthcare workers, can predict the facility of implementation of a future patient safety program and improve the quality of patient care. In addition, this study is the first of its kind to measure the current state of patient SC in Farhat Hached UHC Tunisian hospital. A limitation of the study was the statement bias. In fact, in this study, we use a self-administration questionnaire which can influence respondents behavior and therefore create an over estimated opinion and does not reflect the reality. Another limitation relates to the questionnaire content. Despite the validation of the questionnaire in French and its use by many countries[9, 10], some authors concluded that the survey´s items and dimensions overall are psychometrically sound at the individual, unit, and hospital levels of analysis but that further work is needed in some areas [12].

The results show that all global score rate of ten dimension of patient safety are lower than 75%. The dimensions with the highest positive ratings were for “Frequency of event reporting”, on the other hand, the dimension that had the lowest positive ratings included “Hospital management support for patient safety”. “Frequency of event reporting”, received the highest score. However, it is the most developed dimension among physicians with a score of 84%. The relatively high score obtained from the respondents could be explained by the fact that they expressed their perception instead their real experience. Nevertheless, 66.5 % of respondents felt it necessary to point the person who committed the error and not the problem. It can be attributed to such as factoring lack of open communication and punitive culture of healthcare workers who blamed and punished the responsible person and not the system. Therefore, the staff for fear of punishment, will not report the occurrence of error in their daily practice and limited learning from their mistakes [13]. In fact, developing a blame free climate represented a key strategy to improve error-reporting frequency. This key, associated with promotion of trust in the organization, and using systems approaches to error identification with focus shifted from individuals to processes [3]. So, this non-punitive approach will allow the continuous improvement of the health system´s safety [3]. Teamwork within hospital units is described as developed in most published studies. While team work a cross hospital units is often described as undeveloped. Thus, the study conducted in the United States in 2012 found a score of 80% for team work in the service against 58% for the team work between the services [14]. Similar results were found in surveys conducted in Saudi Arabia with a significantly large difference between team work in the service and between services 57% in Saudi [15].

In our study, the respondents seem unsatisfied with the cooperation among the hospital units and the way these units are coordinated with each other. Indeed, communication within and across hospital units is critical in a healthcare environment as the patient is usually treated by several healthcare practitioners and specialists in multiple settings [16]. Evidence has shown that communication problems Represented one of the major causes of AE [16]. So improving communication can improve the safety of care and ensuring high-quality care [17]. The respondents of this study reported inadequate staffing. Similar scores were found in the USA (58%), lowest in Belgium (38%) and Lebanon (36.8 %) [14, 16, 18]. So this problem concerns most hospitals worldwide. Indeed, in our study, 70% of respondents felt there was a lack of staff to handle the workload. Thus, this lack of staff is a source of weakness in all health care systems. In fact, relationship has been demonstrated between the number of hours of work, care provided by the staff and the mortality rate of patients [16, 19]. Another study explained that increased hours of care provided to patients reduced the duration of stay and complications such as healthcare associated infections [19]. So, intervention would be necessary established in order to adapt and distribute the number of personal health, promoting better care organization, decrease number of hours of health care workers. Therefore, each patient will fully benefit from more quality care. Hospital management must have a big role in creating the culture of patient safety. So management can show its support for patient safety by maintaining open communication, educating personnel, delegating the workers to identify and correct risks, stating that patient safety is a shared responsibility, and providing adequate resources [20]. However, the hospital management support for patient safety is the dimension with the lowest score in our study (32.7%). It is also the most negatively perceived by physicians (13.9%). Similar results were found in many studies conducted in Belgium (35%) and Norway (25%) [18, 21].

## Conclusion

According to survey results, an action plan must be prepared. Administrators, the owner of this process must declare to everyone ownership, ensure open communication between managers-employees and between patients and provide its continuity, identify patient safety threatening situations and delegate responsibility to reduce errors, allocate resources and ensure continuity of training. This action plan must be carried out by both education and practice. In addition, patient safety should be added to the curricula of medical school and nursing. In addition, Patient safety issue must not be an individual effort, but a structured organizational and national aim [22].

### What is known about this topic

- Patient safety is a priority for health systems worldwide and patient safety culture has become a growing concern for all healthcare professionals;
- The success of any intervention with the ultimate aim is securing care and reducing adverse events must go through a developed patient safety culture among healthcare workers;
- The installation of this culture predominates any action for improvement in terms of organization and governance of the healthcare system.

### What this study adds

- This study describes at the first time, in UHC Farhat Hached Sousse- Tunisia the patient security culture among healthcare workers, this first measurement has shown that Patient safety culture is relatively undeveloped among all professionals;
- More precisely, all safety culture dimensions are to develop for paramedical staff while for doctors we note that the perception of “Frequency and reporting adverse events” and “Supervisor/manager expectations and actions promoting safety” seem developed enough (score> 75%);
- The achievement of this study among our health professionals must not have the single purpose to measure the safety culture, to situate and to compare relative to other studies, but must be followed by actions promoting improvement this culture, which is a basis for any successful quality improvement program and safety of care in a health institution.
